# Towards a Process Model of Sustained Attention Tests

**DOI:** 10.3390/jintelligence7010003

**Published:** 2019-01-28

**Authors:** Iris Blotenberg, Lothar Schmidt-Atzert

**Affiliations:** Department of Psychology, Philipps-University of Marburg, Gutenbergstr. 18, 35032 Marburg, Germany; schmidt-atzert@uni-marburg.de

**Keywords:** sustained attention, concentration, process model, experimental test validation, processing speed, reasoning, working memory capacity

## Abstract

Taking up new approaches and calls for experimental test validation, in the present study we propose and validate a process model of sustained attention tests. Four sub-components were postulated: the perception of an item, a simple mental operation to solve the item, a motor reaction, and the shift to the next item. In two studies, several cognitive tasks and modified versions of the d2-R test of sustained attention were applied in order to determine performance in the proposed sub-components. Their contribution for the prediction of performance in sustained attention tests and tests of higher cognitive abilities was assessed. The sub-components of the process model explained a large amount of variance in sustained attention tests, namely 55–74%. More specifically, perceptual and mental operation speed were the strongest predictors, while there was a trend towards a small influence of motor speed on test performance. The measures of item shifting showed low reliabilities and did not predict test scores. In terms of discriminant validity, results of Study 1 indicated that the postulated sub-components were insufficient to explain a large amount of variance in working memory span tasks, in Study 2 the same was demonstrated for reasoning tasks. Altogether, the present study is the first to disentangle sub-components in sustained attention tests and to determine their role for test performance.

## 1. Introduction

Tests of sustained attention (sometimes also referred to as concentration) are among the most popular psychological tests in the domain of cognitive abilities and their validity has been demonstrated in numerous studies [1,2,3,4,5]. Yet, as the field of psychological assessment progresses, calls have been made to take a closer look into the processes that underly test scores and gain a deeper understanding of how these tests scores emerge [6,7,8,9]. For example, the new *Standards for Educational and Psychological Testing* request—in their first chapter—that validity evidence should not only be provided in terms of the test’s internal structure or its relation to other measures, but also in terms of the involved response processes [10]. Regarding sustained attention tests, knowledge about the sub-components that drive performance in these tests would not only increase our understanding of sustained attention tests and what they measure, but it could also be the starting point to investigate well-documented but poorly understood phenomena like the large practice effects in sustained attention tests, respectively. Along these lines, the aim of the present study was to take a first step towards a process model of sustained attention tests. In the following, the construct as well as the characteristics of sustained attention tests are outlined and based on that, a process model comprising four sub-components is derived.

### 1.1. Sustained Attention Tests

Sustained attention may be defined as the ability to achieve and maintain a cognitive state that is characterized by a continuous focus on the relevant task and constant mental effort or as Schweizer [11] (p. 46) put it “the long-term allocation of processing resources to a specific task demand”. This ability is necessary for higher cognitive performances and for a large number of activities in everyday and professional life [12]. Not surprisingly, tests of sustained attention are among the most frequently used cognitive ability tests and they are applied in almost every psychological discipline from business to clinical to traffic psychology [3,4,5]. 

The first tests designed to measure sustained attention were cancellation and mental arithmetic tests and they even go back to the end of the 19th century [13]. Typically, in letter cancellation tests, rows of letters are presented simultaneously and the test-taker is required to look for target stimuli (for example, the letter “a”) and cross out as many of them as possible until the test is over. Similarly, in mental arithmetic tests, many simple equations are presented at the same time and the participants are required to tick as many of the correct equations and to cross out as many of the incorrect equations as possible within a given time limit. The key performance index in these tests is the number of marked items (speed) or the number of correctly marked items (error-corrected speed) [3]. Modern sustained attention tests follow the tradition of these first tests and still use letter cancellation, mental arithmetic, sorting tasks, and other simple tasks [14]. 

Although stimuli and tasks may vary between different sustained attention tests, there are two main features that these tests have in common: First, the stimulus material and tasks are simple [14,15] and second, they share a characteristic presentation mode, i.e., several stimuli are presented simultaneously and participants are required to respond and deliberately shift between them until the test is over [15,16,17]. This so-called self-paced mode constitutes an integral feature of sustained attention tests, because it requires the test-taker to constantly stay on task until the test is over [15,16,17]. In contrast, other attention tests typically apply single stimuli and use fixed inter-stimulus (ISI) or response stimulus (RSI) intervals (also called force-paced mode). Therefore, self-paced sustained attention tests are considered to require higher mental effort, as the test-takers need to continuously allocate processing resources to the task, while force-paced attention tests allow them to take short breaks between successive items [11,15,17,18].

However, while there is a common understanding of the essential features of sustained attention tests, to date, there is no formal model or systematic examination of the sub-components that may drive performance in these tests. It has also been stated that despite its vital role, sustained attention tends to be neglected in the field of cognitive abilities [18,19]. Therefore, in the present paper, we address this knowledge gap and propose and investigate a process model of sustained attention tests. 

### 1.2. A Process Model of Sustained Attention Tests

Imagine a typical sustained attention test that, for example, requires continuous letter or symbol cancellation (e.g., the d2-R [1] or the Frankfurt Attention Inventory [20]) or mental arithmetics (e.g., the Pauli Test [21] or the Revision Test [22]) for a prolonged period of time. First of all, the stimuli need to be perceived and since sustained attention tests present many stimuli at the same time, they impose high perceptual demands [23]. Secondly, the item has to be solved. Although these item-solving processes are not identical for cancellation (e.g., stimulus discrimination and response selection) and mental arithmetic tasks (e.g., mental addition, comparison of solutions, response selection), both share a simplicity and clarity of the cognitive task and a simplicity of the required mental operation. Indeed, previous studies already showed that a variety of sustained attention tests, notwithstanding their differences, correlated highly and loaded on the same factor [2,3]. Thus, we believe it is justified to subsume the different item-solving processes under a common sub-component which we refer to as a simple mental operation. Thirdly, all of these tasks require the execution of some type of motor response (e.g., to tick off, cross out or click on an item) and finally, they demand the deliberate shifting to the next item. Consequently, the test takers’ performance should depend on 1) how quickly the target is perceived (perceptual speed), 2) how quickly the correct reaction to an item is identified (speed of a simple mental operation), 3) how quickly the motor reaction is carried out (motor speed) and additionally, 4) how much time it takes to move on to the next item (item shifting speed; see also References [1,24]). 

Previous studies already investigated some of these proposed sub-components: For example, earlier research demonstrated that the correlation between sustained attention and reasoning measures is partly due to what the authors called shared perceptual and discrimination processes [23]. Moreover, it was shown that motor speed played a role for performance in a variety of sustained attention tests [3,22,25]. Finally, the intentional, self-paced shifting between items has repeatedly been mentioned as an important process in these tests [15,16,17,18,26]. The purpose of the present study is to extend these findings by exploring the role of the four proposed sub-components for performance in sustained attention tests. Moreover, while we presume these sub-components to be the main predictors of performance in sustained attention tests, in the following, we argue that tests of higher cognitive abilities should require more and also more complex mental operations beyond the sub-components of our process model of sustained attention tests. 

### 1.3. Discriminant Validity: Processes in Higher Cognitive Abilities

Sustained attention is typically regarded as a prerequisite of higher cognitive performances [12]. These more complex cognitive abilities like reasoning (considered the best indicator of g [27]) or working memory capacity (purportedly involved in a wide range of complex intellectual abilities, see Reference [28] for a review) involve additional and also more complex sub-processes or as Hunt [29] (p. 457) put it “…tests of intelligence…seem to require the orchestration of several different functions”. Sternberg [30] identified several component processes in analogical reasoning, involving encoding (perception and storage of attributes), inference (discovering and storage of the relation), mapping (discovering the relation between the first and the second half), application (of the analogy to each answer option), justification (determining the superior answer if none fits perfectly), and finally, a response. Thus, while speeded reasoning tasks also require the fast perception of an item and a quick motor reaction, they additionally demand complex, iterative mental operations not covered by our process model of sustained attention tests.

Similarly, tasks of working memory span demand additional cognitive processes beyond the proposed sub-components. These tasks require the processing of new information and the storage and retention of information (that is no longer present) at the same time [31,32,33], involving processes like rehearsal, maintenance, memory updating, and controlled memory search [33,34]. Hence, while these tasks of higher cognitive abilities share some of the proposed sub-components, they additionally demand more complex mental operations not included in our process model of sustained attention. We would therefore, in terms of discriminant validity, expect our sub-components to be insufficient to explain a large amount of variance in these tests. 

### 1.4. Aims of the Present Research

The main objective of the present study was to provide and validate the proposed sub-components of sustained attention tests by firstly, assessing the speed in these sub-components and secondly, examining to what extent these sub-components predicted performance in conventional sustained attention tests. The inspection time task [35], which is considered to impose mainly perceptual demands [36,37,38], was selected as a measure of perceptual speed. To assess the speed of the item-solving process, a modified version of the d2-R test of sustained attention was created that allowed the measurement of reactions to single items. Additionally, the simple reaction time task was applied as a prototypical measure of motor speed [39,40,41]. Furthermore, in the modified d2, the response-stimulus interval between successive items was varied within subjects to assess item shifting. It was anticipated that shorter RSI, which require the test-taker to immediately shift between items, would impede performance compared to longer intervals [42,43]. 

We then specified a model in which the sub-components predicted performance in sustained attention tests as well as tests of higher cognitive abilities. It was expected that the speed in the four sub-components would be significantly related to performance in sustained attention tests, that is, the faster the test taker perceived, solved, responded to the item, and shifted towards the next item, the higher the test score. In contrast, in terms of discriminant validity, more complex cognitive abilities like reasoning or working memory capacity should involve additional and also more complex processes not covered by our process model of sustained attention tests [30,33]. Thus, we would expect the proposed sub-components to explain only a limited amount of variance in these tests.

## 2. Study 1

### 2.1. Materials and Methods

#### 2.1.1. Participants

One-hundred-and-three undergraduates of the Philipps University of Marburg gave informed consent prior to taking part in the study, participated voluntarily, and received partial course credit in exchange. The mean age was 23 years (*SD* = 5.2, range = 18–58) and the majority was female (68%). Many studied psychology (43%), followed by economics (11%), educational sciences (11%), and other subjects. They had a mean educational training of 3.5 semesters (*SD* = 1.7). The participants reported a normal or corrected-to-normal vision. 

#### 2.1.2. Procedure

Participants were tested in groups of two to five in a laboratory. Each test session took about four hours including three ten minutes breaks. First, a pre-experimental questionnaire, the inspection time task, the simple reaction time task, the modified version of the d2 and the Revision Test were applied. After a break, they were followed by the electronic version of the d2-R and three reasoning tasks. The second break was followed by three verbal sustained attention tasks, the simple reaction time task (second administration), the modified d2 (second administration), and three working memory span tasks. After the third and final break and irrelevant for the present study, further cognitive tests and a final questionnaire were administered. 

#### 2.1.3. Measures

Several cognitive tasks were applied to test the proposed process model of sustained attention tests. As the overall aim was to gain a better understanding of the processes involved in these tests, we applied three sustained attention tests, namely the d2-R [1], the Revision Test [22], and three verbal subtasks from the Berlin Intelligence Structure (BIS) Test [44] (sustained attention and processing speed, as measured in the BIS, are conceptually similar and have been shown to measure the same construct [3,45]). These tasks were selected based on recommendations by Schmidt-Atzert et al. [3] and covered figural, numerical, and verbal demands [3]. For the tasks that were selected or created to assess the speed in the proposed sub-components of sustained attention tests, the d2-R served as a template. For example, a modified d2 was applied to measure operation speed. Finally, due to their good psychometric properties, figural, numerical and verbal reasoning tasks of the Intelligence-Structure-Test 2000 R [46] and figural, numerical, and verbal complex span tasks [47] were selected as measures of discriminant validity.

##### Conventional Sustained Attention Tests

Test d2-R Electronic Version [1]. In the d2-R, the task was to select the letter “d” with two marks out of “d” s and “p” s with one to four marks. Fourteen computer pages were applied, each consisted of 60 letters and was presented for 20 s (4.40 min in total, plus instructions). The dependent variable was the number of correctly marked items minus the number of confusion errors (error-corrected speed). 

Revision Test [22]. In this paper–pencil test, the task was to quickly assess whether a simple equation was correct, to tick the item if it was and to struck it out if it was not. The tests consisted of 15 lines with 44 items and 30 s per line (7.30 min in total, plus instructions). The number of correctly marked equations was assessed. 

Berlin Intelligence Structure (BIS) Tasks of Verbal Processing Speed [44]. Three paper-pencil tasks of the Berlin Intelligence Structure Test were administered to measure verbal sustained attention. The first task “classification of words” (CW) was to strike out as many plants as possible from a list of 100 words within 30 s. In the second task “uncompleted words” (UW), 57 incomplete words were presented on a sheet of paper and the missing letters had to be filled in within 50 s. In the third task “part-whole” (PW), a list of 60 words was presented for 40 s and words with a certain semantic relation (a word that was part of another, e.g., month and year) had to be marked. Performance indices were the number of correct responses. 

##### Tasks Assessing the Sub-Components

Perceptual Speed: The Inspection Time Task. In this task, exposure time of a pi-shaped figure with two legs of markedly different lengths was shortened adaptively. Critically, this task was about the shortest exposure time necessary to perceive and correctly indicate which of the two lines was longer, not about reaction times. It is considered a primarily perceptual task and was therefore selected as a measure of perceptual speed [36,37,38]. The present version of the task was programmed in E-Prime 2.0 [48]. On each trial, a white fixation cross was displayed on a black screen (1000 ms). Then, a white Pi-shaped figure with a shorter (3.5 cm) and a longer (4.5 cm) leg was presented against a black background and afterwards covered by a backward mask (300 ms). Subjects indicated which leg was longer by pressing “c” (left index finger, the key was colored red) or “m” (right index finger, the key was colored green) on a German QWERTZ computer keyboard. Stimulus exposure time was varied using a staircase procedure, i.e., four correct responses led to a shortened exposure time while an incorrect response led to a prolonged exposure time. After three practice trials, the staircase procedure started with an exposure time of 157 ms and decreased or increased in steps of 66 ms (at the beginning) to 16.5 ms (as the experiment proceeded). The experiment ended after 15 reversals (i.e., exposure time which decreased before suddenly increased or vice versa) or 96 trials. The individual inspection time was assessed as the shortest exposure time of the stimulus to which the participant managed to correctly react to for four times in a row.

Mental Operation and Item Shifting Speed: The Modified d2. This task was programmed in E-Prime 2.0 [48]. In each trial, three letters were presented simultaneously until the participant made a response. The task was to decide whether there was a d2 among them and press “m” (right index finger, the key was colored green) for a target and “c” (left index finger, the key was colored red) for a nontarget on a German QWERTZ computer keyboard. The number of targets and nontargets was counterbalanced, as was the position of the d2 and the congruence of flanking stimuli (for targets, i.e., whether there were one, two or three d2). Altogether, the task comprised 432 experimental plus 10 practice trials (RSI of 500 ms) and took about 20 minutes. Mean reaction times (RT) served as indicators of mental operation speed^1^. However, the modified d2 also required efficient perceptual and motor processes. Therefore, overlapping cognitive processes between the different indicators of the sub-components were taken into account when specifying structural equation models in the data analysis. 

Critically, in the modified d2, the response–stimulus interval (RSI) was varied (blockwise to avoid sequential effects due to mixed RSI [49]) in order to assess item shifting speed. The first experimental block consisted of 18 stimuli and included an RSI of 1000 ms. In the following blocks, the RSI was decreased in steps of 100 ms until it was 0. The last experimental block used an RSI of 4000 ms. From there, the pattern of RSI started all over again. It was expected that short RSI impede performance and that participants’ reactions become faster and more accurate as the RSI gets longer [42,43] until the ideal time interval is approached. From there, performance should not benefit from further extension of the RSI [49,50]. Our rationale for determining item shifting speed was that, due to this relationship between RSI and RT, fast reactions should have been preceded by a sufficiently long RSI. Therefore, as an estimator of the time needed for an item shift, the average of RSI preceding the fastest 30% of correct reactions was determined. 

Motor Speed: A Simple Reaction Time (SRT) Task. Participants were instructed to press the button “c” (Block 1, 20 trials, left index finger, the key was colored red) and afterwards the button “m” (Block 2, 20 trials, right index finger, the key was colored green) as fast as possible as soon as a black dot (2 × 2 cm) appeared on screen. The dot was presented until a response was made and the next dot appeared after 1000 ms (+/−100 ms jitter). Altogether, the SRT task consisted of 40 experimental plus 10 practice trials. Mean RT in the SRT task served as indicators of motor speed, because this task is considered a prototypical measure of motor performance [39,40,41]. However, it also imposes basic perceptual demands which will be taken into account in the data analysis.

##### Divergent Validity: Reasoning and Working Memory Span Tasks

I-S-T 2000 R Electronic Version [46]. Figural reasoning was assessed using the task “matrices”. Participants had to discover the rule underlying the placement of three figures and choose the missing figure out of five response options. In the numerical reasoning task “number series”, participants had to identify the underlying rule in a row of seven numbers and fill in the next number in line (time limit for both tasks: 10 min for 20 items). In the verbal reasoning task “verbal analogies”, participants had to find the relation between two words and find a word that has a similar relation to the third word out of five response options (time limit: 7 min for 20 items). The dependent variable in each task was the number of correct responses.

Complex Span Tasks. Three complex span tasks were applied to assess working memory capacity: Operation span, reading span, and symmetry span [47]. These tasks were similarly structured: They started with a series of practice trials in which 1) a storage task, 2) a processing task, and 3) the combination of the storage and the processing task were practiced. Afterwards, the test trials started in which the storage and the processing tasks were combined. In order to force the participants to focus on the processing task and not only on the recall of the elements, the time for one processing task in the test trials was limited to 2.5 standard deviations of the mean time for a processing task in the practice trials. Additionally, participants received feedback of the percentage of the correctly answered processing tasks. They were informed that their data would only be used if at least 85% of the processing tasks were answered correctly. 

In the “symmetry span” task, participants were presented with a set of figures that were either symmetrical or asymmetrical down the vertical axis. After each figure, a 4 × 4 matrix was shown and one square of this matrix was colored red. The position of the red square in the matrix had to be recalled at the end of the set. The numeric version of this group of tasks was an “operation span” task. Participants were presented with a set of arithmetic operations and the task was to judge whether an equation was correct or not. After each arithmetic operation, a letter was presented that had to be recalled at the end of the set. In the “reading span” task, participants were presented with a set of sentences and had to judge whether each sentence was sensible or not. After each sentence, a letter was presented that had to be recalled at the end of the set. Set sizes ranged from 3–5 in the symmetry span task and from 4–6 in the operation and reading spans tasks. Each set size was administered two times (six items comprising 30 symmetry-square, equation-letter or sentence-letter pairs, respectively). The absolute score for each of the three tasks was the number of trials that were recalled in the correct order and without error.

### 2.2. Data Preprocessing

For the RT analysis of the modified d2, errors were excluded and based on the correct trials, z-values were calculated for each participant in each block; trials with a RT above *z* = 2.5 and below *z* = −2.5 were discarded as outliers. The same cut-offs were applied in the SRT task. For the inspection time task, one participant who stated to have pressed the buttons randomly in the post-experimental questionnaire and whose inspection time was above *z* = 4 was excluded. Regarding the modified d2, more than 30% of incorrect responses led to an exclusion of the participant’s data in this task (one participant). For the working memory span task, participants whose accuracy in the processing tasks was below 85% were excluded (see recommendations in Reference [47]; two participants for the verbal, numeric, and figural version). 

### 2.3. Analysis Strategy

First, as a precondition to assess a potential item shifting component, it was investigated whether short response–stimulus intervals between successive stimuli indeed impeded performance compared to longer response–stimulus intervals as reported in earlier studies [42,43]. Additionally, the descriptive statistics and reliability of the sub-components and cognitive ability tasks were inspected. Finally, to assess the predictive power of the process model, we specified structural equation models in which the four sub-components of perceptual, mental operation, motor and item shifting speed predicted performance in the employed sustained attention tests (convergent validity) and in the reasoning and working memory tasks (discriminant validity). 

### 2.4. Results

#### 2.4.1. Calculating an Indicator of Item Shifting Speed

**Figure 1:**
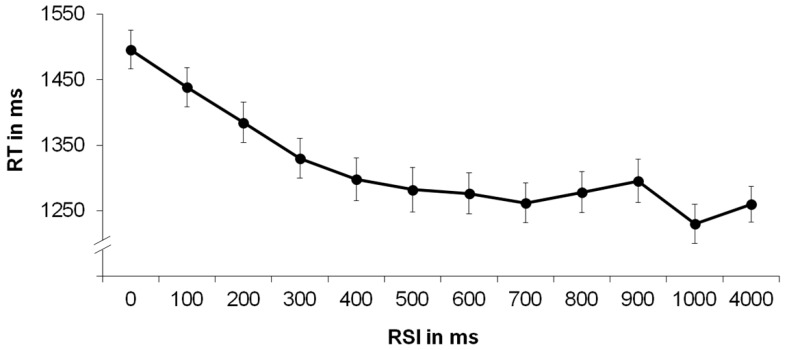
Mean reaction time (RT) (in ms) as a function of the duration of the response–stimulus interval (RSI) (in ms).

The relationship between RSI and RT is depicted in Figure 1. As expected, a repeated-measures ANOVA with the within-subjects factor RSI yielded a significant effect, *F*(7.939, 809.754) = 55.810, *p* < .000, *η^2^p* = .354^2^, indicating increasingly shorter RT as the RSI became longer.

Post-hoc tests using the Bonferroni correction revealed that increasing the RSI from 0 to 100 ms, from 100 to 200 ms and up until 300 ms elicited a significant decrease in RT, respectively (*p* < .05). These results suggest that the length of the RSI influenced RT and that very short RSI impeded performance. This relationship between RSI and RT was utilized to determine the individual time necessary for an item shift by selecting the fastest 30% of reactions and calculating the average of the preceding RSI. 

#### 2.4.2. Descriptive Statistics and Reliability

Means, standard deviations, and reliability estimates of the study measures are presented in Table 1, the correlation matrix is presented in Appendix A. Reliability estimates were high for the modified d2 (*r_tt_* = .89 (in retest)–.99 (Cronbach’s α)) but low for the SRT task (*r_tt_* = .55 (in retest)–.60 (Cronbach’s α)) and poor for the indicator of item shifting speed (*r_tt_* = .41 (in retest)–.51 (Cronbach’s α)). All applied sustained attention tests showed good or excellent reliabilities (*r_tt_* = .80 (in retest)–.98 (Cronbach’s α)). Additionally, Cronbach’s α for the reasoning and working memory span tasks ranged from low (*r_tt_* = .56) for figural working memory span) to very satisfactory (*r_tt_* = .90 for numerical reasoning).

#### 2.4.3. Structural Equation Modeling

The contribution of the subcomponents of perceptual, mental operation, motor and item shifting speed for the prediction of performance in sustained attention tests, reasoning and working memory span tasks was assessed using Amos 24.0 [51]. Prior to the structural equation modeling, missing values were imputed using the expectation-maximization (EM) algorithm [52].

For the modified d2 (mental operation speed), motor (simple reaction time task), and item shifting speed, trials were randomly assigned to one of three indicators, to assure identifiability of the model [53]. Regarding the adaptive inspection time task, since it cannot be spitted, the reliability estimates for adults as reported in a meta-analysis [54], *r_tt_* = .73, was incorporated in the model via the error term [55]. All study measures were converted so that high values reflected better performance.

In the model, the modified d2 was regressed on perceptual speed (measured as inspection time) and motor speed (measured as simple reaction time) to account for shared perceptual and motor processes and to extract a residual of the modified d2 that would capture mental operation speed. Similarly, as the simple reaction time task, which was applied as a measure of motor speed, also imposed basic perceptual demands, it was regressed on perceptual speed (measured as inspection time) to derive a residual that would represent the sub-component of motor speed. Performance in the applied sustained attention, reasoning and working memory span tests was regressed on the so-obtained sub-components of perceptual, mental operation, motor and item shifting speed. 

##### Predicting Performance in Sustained Attention Tests

**Figure 2:**
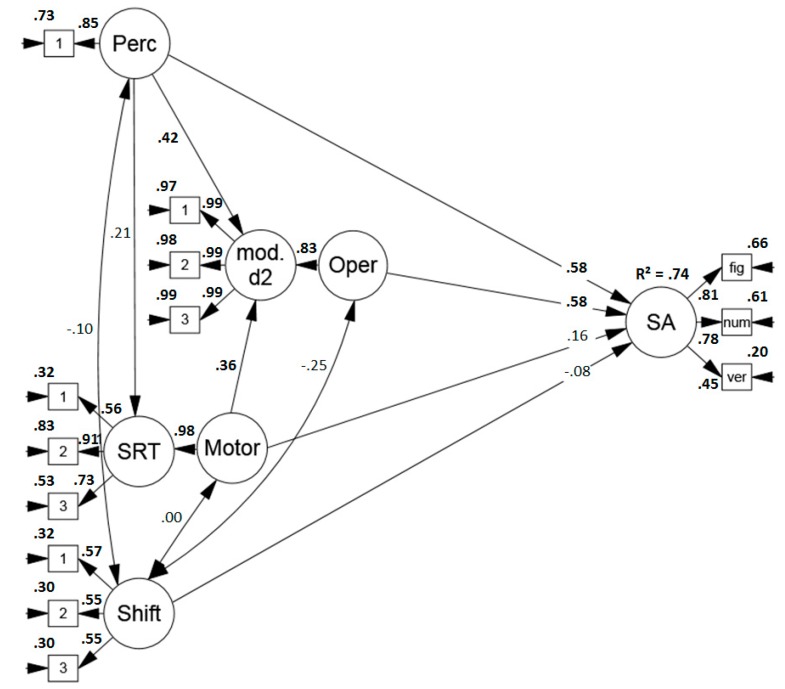
Standardized solution of the model predicting performance in sustained attention tests (convergent validity) based on the proposed process model of sustained attention tests. Significant results are printed in boldface. Perc = perceptual speed, mod. d2 = modified d2, Oper. = operation speed, SRT = simple reaction time task, Motor = motor speed, Shift = item shifting speed, SA = sustained attention, fig = figural task, num = numerical task, verb = verbal task.

First of all, a model was specified to examine the sub-components’ contribution for the prediction of performance in sustained attention tests. The model (see Figure 2) indicated a good fit (*χ*2 (56) = 58.645, *p* = .379, RMSEA = .022 (.000, .066), CFI = .997, SRMR = .049) and the sub-components explained a large amount, namely 74%, of sustained attention test variance. Perceptual and mental operation speed were the main predictors. Descriptively, there was a small influence of motor speed but it failed to reach significance (*p* = .13). The indicator of item shifting speed did not predict performance.

##### Predicting Performance in Sustained Attention, Reasoning and Working Memory Span Tasks

**Figure 3:**
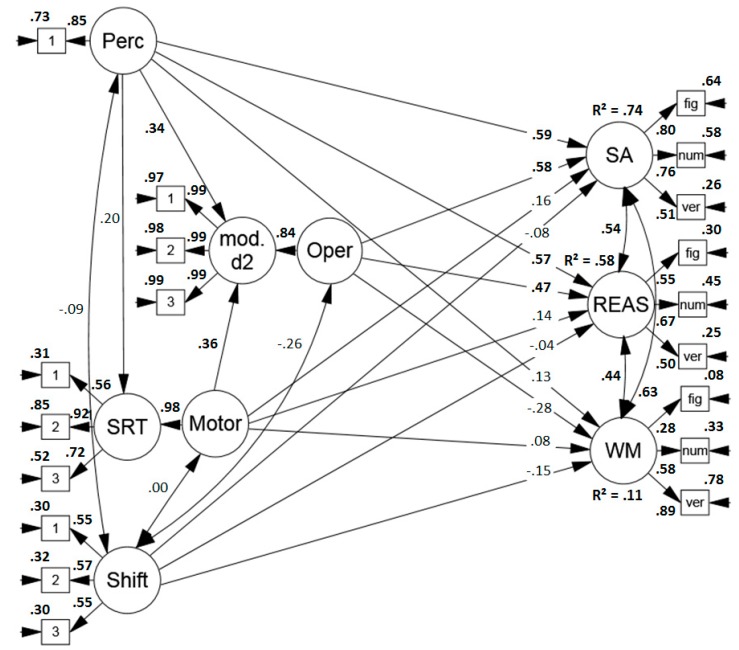
Standardized solution of the model predicting performance in sustained attention tests (convergent validity), reasoning, and working memory span tasks (discriminant validity) based on the proposed process model of sustained attention tests. Significant results are printed in boldface. Perc = perceptual speed, mod. d2 = modified d2, Oper = operation speed, SRT = simple reaction time task, Motor = motor speed, Shift = item shifting speed, SA = sustained attention, REAS = reasoning, WM = working memory span, fig = figural task, num = numerical task, verb = verbal task.

Secondly, the model was extended to assess the predictive power of the sub-components for performance in tests of higher cognitive abilities. The model (see Figure 3) revealed an acceptable to good fit (*χ*2 (132) = 161.630, *p* = .386^3^, RMSEA = .047 (.011, .070), CFI = .974, SRMR = .073). Surprisingly, the correlation between sustained attention and reasoning performance was higher than reported in earlier studies (for a meta-analysis, see Reference [56] which reports a mean correlation of *r* = .29). Moreover, unexpectedly, the process model predicted performance in reasoning tests well and explained 58% of the test variance. Not only perceptual speed, but also the speed of the relatively simple mental operation in the modified d2 was a good predictor of reasoning performance. Finally, as anticipated, the four sub-components explained only a modest amount of variance, namely 11% in working memory span performance.

### 2.5. Discussion

In Study 1, individual differences in the sub-components explained 74%, and thus, a large amount of variance in sustained attention tests. More specifically, two components, namely perceptual and mental operation speed showed to be strong predictors of test performance, while there was a small, but not significant (*p* = .13) influence of motor speed. 

**Figure 4:**
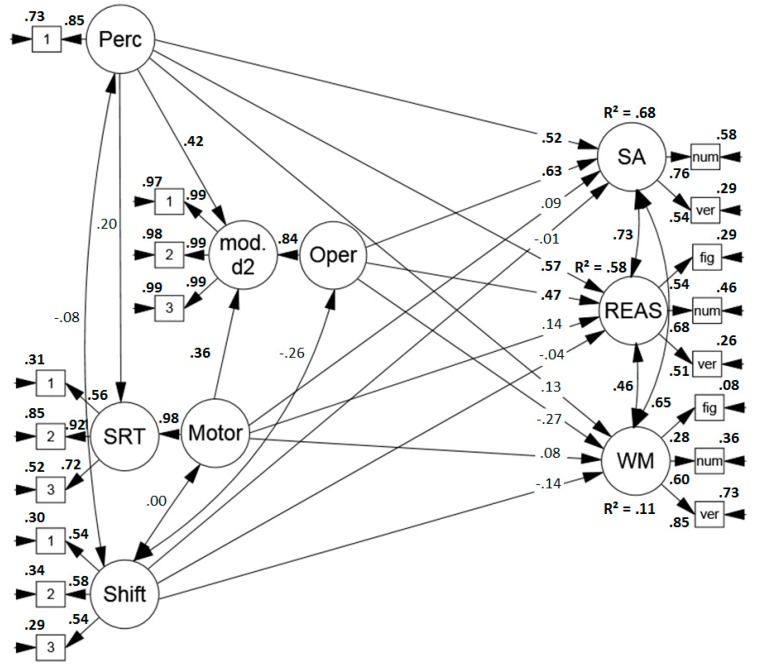
Standardized solution of the model predicting performance in sustained attention tests (convergent validity, without the d2-R as dependent variable), reasoning and working memory span tasks (discriminant validity) based on the proposed process model of sustained attention tests. Significant results are printed in boldface. Perc = perceptual speed, mod. d2 = modified d2, Oper = operation speed, SRT = simple reaction time task, Motor = motor speed, Shift = item shifting speed, SA = sustained attention, REAS = reasoning, WM = working memory span, fig = figural task, num = numerical task, verb = verbal task.

Yet, it is conceivable that these results may partly be due to an overlap between the modified d2 and the d2-R test of sustained attention that served as one of the dependent variables. Therefore, another model was run in which only the Revision Test and the verbal subtasks of the BIS served as indicators of sustained attention test performance (see Figure 4). The model showed a sufficient to good fit (*χ*2 (115) = 141.459, *p* = .358^4^, RMSEA = .047 (.005, .072), CFI = .975, SRMR = .071). Altogether, the sub-components explained 68% of the variance in sustained attention performance and again, perceptual and mental operation speed were the strongest predictors. Thus, the explanatory power of the proposed process model for performance in sustained attention tests was not only due to an overlap between the applied tasks. 

However, while it was demonstrated that the process model successfully predicted a large amount of variance in sustained attention tests, there was no relationship between item shifting speed and performance in sustained attention tests. On the one hand, this is surprising, as theoretical considerations and earlier research point towards a special significance of the self-paced presentation mode of sustained attention tests [5,15,16,17], which suggests that a test-taker who succeeds in the fast, deliberate shifting between items should also achieve a higher score in sustained attention tests. On the other hand, we did not succeed in reliably measuring this proposed sub-component with a retest-reliability of .41 and a split-half reliability of .51. We can think of two possible explanations for this: first, item shifting is a flexible process which varies from trial to trial, and thus, represents a state rather than a cognitive ability, or second, our operationalization and measurement of item shifting was inadequate. In order to examine the latter, we took a different approach to investigate item shifting in Study 2. 

Moreover, the process model ex hypothesis failed to predict performance in working memory span tasks. However, regarding reasoning performance, not only perceptual speed but also mental operation speed explained a large amount of variance in these tests. While it was not surprising that perceptual processes played a role in reasoning tests (see also Reference [36]), there was the expectation that the simple mental operation required in sustained attention tests would be insufficient to account for the complex processes involved in reasoning. We identified two potential reasons for this finding: First, sustained attention and reasoning tests were unusually highly correlated (*r* = .58) in the present study, whereas earlier studies reported moderate correlations between these cognitive abilities [3,56]. A reason for this high correlation might have been the relatively late administration (right after several sustained attention tests and more than one hour into the session) of the reasoning tests in the middle of a potentially exhausting, four-hour long test battery of cognitive ability tests. That means, the long test session might have changed the validity of the reasoning tests so that they captured sustained attention abilities especially well. Second, there is much evidence that the complexity of a task and thereby its correlation with reasoning increases with the number of relevant stimuli [57,58,59]. Therefore, the modified d2 with its three simultaneously relevant stimuli may have been too complex, and thus, might have become a good indicator of reasoning performance.

In Study 2, we address the main issues of Study 1 by (1) operationalizing the proposed item shifting ability differently, (2) presenting the reasoning tests at the beginning of the test battery, and (3) lowering the complexity of the modified d2 in order to measure mental operation speed more adequately.

## 3. Study 2

### 3.1. Materials and Methods

#### 3.1.1. Participants

One hundred students (72% female) voluntarily participated in Study 2 and received partial course credit in exchange. Their mean age was 22.9 years (*SD* = 4.6, range = 18–40) and they had studied 3.2 semesters (*SD* = 2.4) on average in fields like psychology (42%), educational sciences (18%) or economics (9%). Participants gave informed consent in accordance with the Declaration of Helsinki prior to participation. 

#### 3.1.2. Procedure

Each test session took about two hours including two ten-minute breaks. It started with a pre-experimental questionnaire, followed by three reasoning tasks. After a break, the inspection time task, the simple reaction time task, the modified d2 and the Revision Test were applied. After another break, the electronic version of the d2-R and the verbal sustained attention tests were administered, followed by a second exposure of the inspection time task, the simple reaction time task, the modified d2 and a final short questionnaire. 

#### 3.1.3. Measures

For the assessment of the sub-components of sustained attention tests, another modified d2 was created in order to overcome limitations of the first study. Thus, the assessment of mental operation speed and item shifting differed from Study 1 and is described below. Regarding the assessment of sustained attention and reasoning performance, the same tests were applied as in Study 1. 

##### Tasks Assessing the Sub-Components

Mental Operation Speed and Item Shifting: The modified d2. Similarly, as in Study 1, the rationale for determining item shifting costs was that responses are faster and more accurate when the length of the RSI is appropriate, whereas too short RSI impede performance [42,43]. In Study 2, item shifting costs were determined as the difference in RT in the conditions with (force-paced) and without an RSI (self-paced). 

In the modified version of the d2, the pace of the task was manipulated (force-paced vs. self-paced) and as part of another study, the stimulus arrangement was varied. In the first two blocks, one stimulus was presented at a time and participants had to decide whether the relevant letter was a d2 (response: “m”, right index finger) or not (response: “c”, left index finger). In Blocks 3 and 4, three stimuli were presented at a time, but only the one in the center was relevant. Critically, after the response, there was an RSI of 500 ms in Blocks 1 and 3 (force-paced conditions) and this RSI was removed in Blocks 2 and 4 (self-paced conditions). Each block consisted of 80 stimuli plus 10 practice trials (15 min in total). 

Mean RT of the first two blocks of the modified d2 served as indicators of mental operation speed. As a measure of item shifting costs, the difference in RT of the conditions with (Block 1 and 3) and without an RSI (Block 2 and 4) was assessed. That means, we investigated individual differences in the extent to which RT increased as the participants were required to continuously react to stimuli (self-paced conditions) compared to conditions that included short breaks between successive stimuli (force-paced conditions).

### 3.2. Data Preprocessing

With regard to data cleaning, the same procedure and criteria were applied as in Study 1. For the inspection time task, three participants who mentioned difficulties with the task and whose inspection time was above *z* = 4 were excluded. For the modified d2, accuracy below 70% in the task led to the exclusion of the participant’s data (three participants). 

### 3.3. Analysis Strategy

First, as a precondition to determine item shifting costs, it was assessed whether the self-paced mode, which required the deliberate shifting between items, impeded performance compared to the force-paced mode, which allowed short intervals between successive items. Additionally, the descriptive statistics of the study variables were inspected and it was examined whether they showed sufficient reliabilities. Finally, structural equation models were specified in which the four sub-components predicted performance in different sustained attention tests (convergent validity) and reasoning tests (discriminant validity). 

### 3.4. Results

#### 3.4.1. Calculating an Indicator of Item Shifting Costs

As a precondition for determining item shifting costs, it was assessed whether RT differed significantly between the force-paced and the self-paced blocks. This was confirmed, *t* (96) = 14.980, *p* < .001, indicating longer RT in the self-paced blocks (*M* = 704 ms, *SD* = 92.5) than in the force-paced blocks (*M* = 628 ms, *SD* = 86.7). Individual item shifting costs were calculated by first log-transforming response latencies and then subtracting mean RT in the force-paced from mean RT in the self-paced blocks. Log-transformed differences between times equal ratios. We were interested in the individual’s relative performance decline or improvement as a function of the self-paced vs. force-paced mode (see also References [32,60]).

#### 3.4.2. Descriptive Statistics and Reliability

Table 2 provides means, standard deviations, and reliability estimates of the study variables. The correlations of the study measures are presented in Appendix B. Reliability was sufficient to high for RT measures of the modified d2 (*r_tt_* = .68 –.89 (in retest), *r_tt_* = .92 –.97 (split-half)) and tests of sustained attention (*r_tt_* = .83 –.98 (Cronbach’s alpha)), but low for the inspection time task (*r_tt_* = .58 (in retest)), the SRT task (*r_tt_* = .57 (in retest) –.75 (split-half)) and for item shifting costs (*r_tt_* = .46 (in retest) –.80 (split-half)). Cronbach’s alpha was high for the numerical reasoning test (*r_tt_* = .83) and considerably lower for the figural (*r_tt_* = .57) and verbal (*r_tt_* = .75) reasoning task.

#### 3.4.3. Structural Equation Modeling

For the modified d2 (mental operation speed), the simple reaction time task (motor speed), and item shifting, trials were randomly assigned to one of three indicators. Regarding the inspection time task, its retest-reliability in Study 2, *r_tt_* = .58, was incorporated in the model via the error term [55]. All study measures were converted so that high values reflected better performance.

##### Predicting Sustained Attention

**Figure 5:**
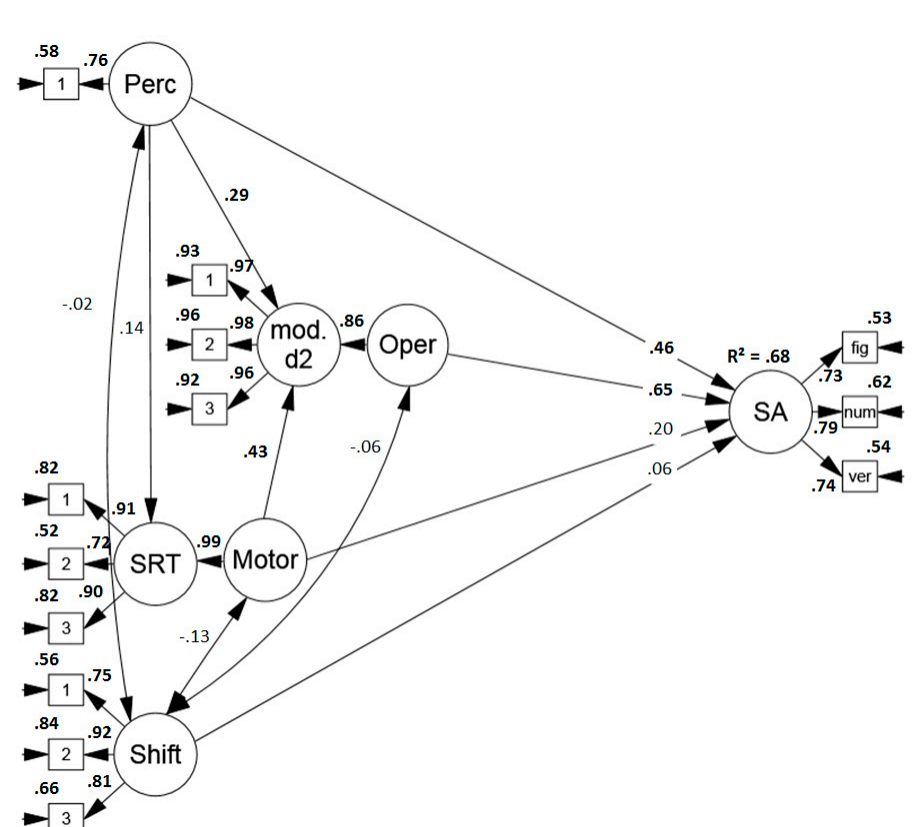
Standardized solution of the model predicting performance in sustained attention tests (convergent validity). Significant results are printed in boldface. Perc = perceptual speed, mod. d2 = modified d2, Oper = operation speed, SRT = simple reaction time task, Motor = motor speed, Shift = item shifting speed, SA = sustained attention, fig = figural task, num = numerical task, verb = verbal task.

First, it was assessed to what extent the sub-components predicted performance in the applied sustained attention tests. The model (see Figure 5) revealed a good fit (*χ*2 (56) = 84.824, *p* = .156^5^, RMSEA = .072 (.038, .102), CFI = .970, SRMR = .079) and the sub-components predicted 68% of the variance in sustained attention tests. Perceptual and mental operation speed were the strongest predictors and there was a trend towards a minor influence of motor speed (*p* = .08), whereas item shifting costs did not predict test scores.

##### Predicting Sustained Attention and Reasoning Test Performance

**Figure 6:**
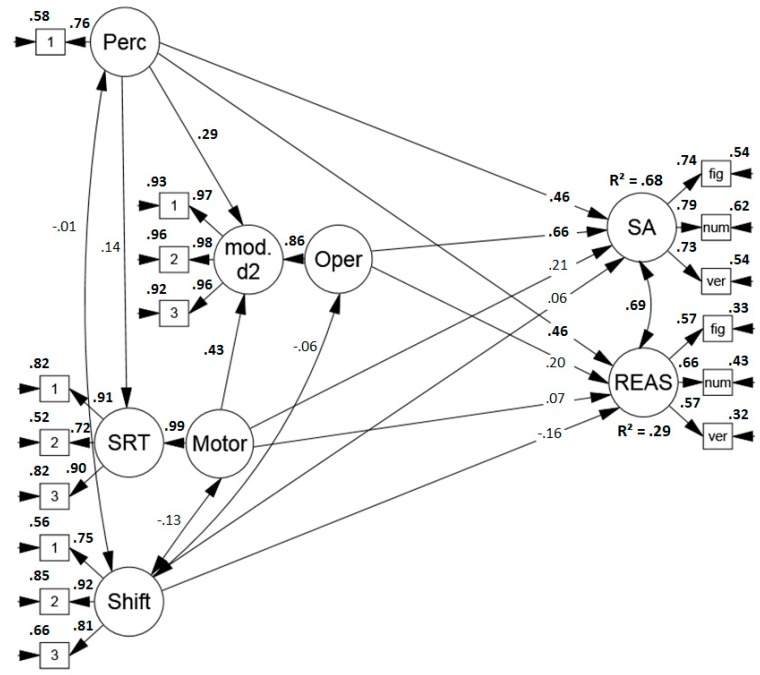
Standardized solution of the model predicting performance in sustained attention tests (convergent validity) and performance in reasoning tests (discriminant validity). Significant results are printed in boldface. Perc = perceptual speed, mod. d2 = modified d2, Oper = operation speed, SRT = simple reaction time task, Motor = motor speed, Shift = item shifting speed, SA = sustained attention, REAS = reasoning, fig = figural task, num = numerical task, verb = verbal task.

Second, the model was extended to examine the sub-components’ contribution for the prediction of performance in reasoning tasks. The model (see Figure 6) indicated an acceptable to good fit, (*χ*2 (90) = 124.366, *p* = .197^6^, RMSEA = .062 (.032, .087), CFI = .967, SRMR = .079). As expected, the process model explained a modest amount of variance in reasoning performance, namely 29%, with perceptual speed being the strongest predictor.

### 3.5. Discussion

Similarly, as in study 1, the proposed sub-components revealed a high predictive value for performance in SA tests. Overall, the process model explained 68% of the variance in sustained attention tests. Similarly, as in Study 1, perceptual and mental operation speed were the strongest predictors and there was a trend towards a small influence of motor speed (*p* = .08).

**Figure 7:**
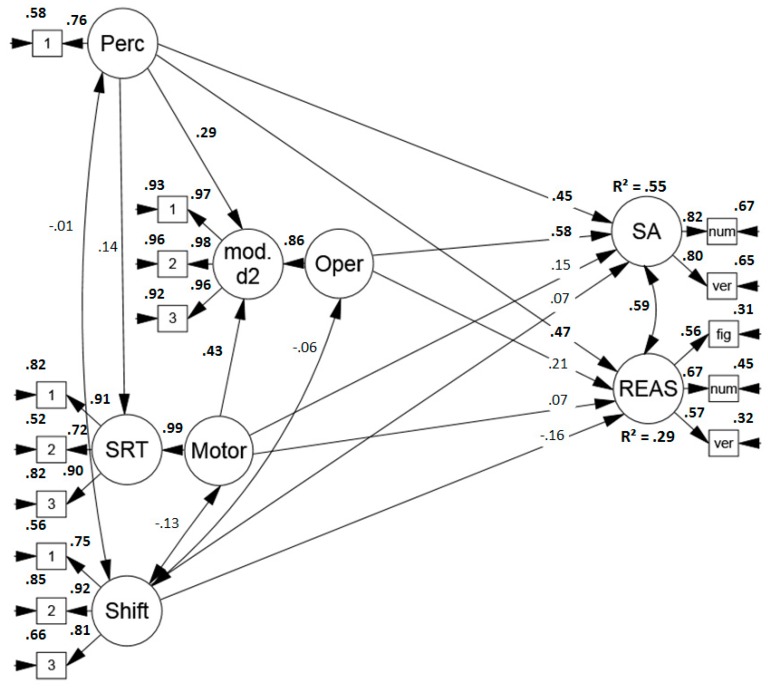
Standardized solution of the model predicting performance in sustained attention tests (convergent validity, without the d2-R as dependent variable) and performance in reasoning tests (discriminant validity). Significant results are printed in boldface. Perc = perceptual speed, mod. d2 = modified d2, Oper = operation speed, SRT = simple reaction time task, Motor = motor speed, Shift = item shifting speed, SA = sustained attention, REAS = reasoning, fig = figural task, num = numerical task, verb = verbal task.

However, as the test material of the modified d2 and the d2-R test of sustained attention overlapped considerably, we conducted another analysis and excluded the d2-R test of sustained attention as the dependent variable (see Figure 7). Again, the model fit was good (*χ*2 (76) = 94.676, *p* = .349^7^, RMSEA = .050 (.000, .080), CFI = .981, SRMR = .070). Altogether, the process model explained less but still a majority, namely 55% of the variance in sustained attention test performance. Still, perceptual and mental operation speed were the strongest predictors.

This time, we took another approach to operationalize item shifting by investigating item shifting costs, that is the decline in performance in a self-paced (without RSI) compared to a force-paced (with an RSI) condition of the modified d2. Although the reliability of the item shifting measure (*r_tt_* = .46 (in retest)–.80 (Cronbach’s alpha)), was higher than in study 1 (*r_tt_* = .41 (in retest)–.51 (split-half)), it again failed to predict performance in sustained attention tests. Note though that RT difference scores are typically lower in reliability than mean RT [61]. Overall, results are highly similar to Study 1 with the notable exception that, in accordance with our hypothesis, the process model explained a considerably smaller amount of variance in reasoning performance. In Study 2, only 29% of the variance in reasoning tests could be explained by the proposed process model, thus supporting discriminant validity of the postulated sub-components with regard to reasoning ability. 

## 4. General Discussion

In the present study, a generic process model of sustained attention tests was provided based on earlier research on the characteristics of this group of tests. It comprises four essential sub-components: item perception, a simple mental operation, a motor response, and item shifting, hereby taking into account the variety of sustained attention tests. Furthermore, this model was validated by determining the speed in the proposed sub-components and it was demonstrated that two main sub-components, namely perceptual and mental operation speed, successfully predicted a large amount of variance in these tests. Moreover, regarding the third sub-component, motor speed, there was a trend towards a small influence on performance in sustained attention tests. In line with our expectations, the results indicate that while our process model predicted performance in sustained attention tests well, it was by and large insufficient to explain a large amount of variance in tasks of higher cognitive abilities like reasoning or working memory capacity.

### 4.1. The Role of the Sub-Components for Performance in Sustained Attention Tests

Most importantly, the proposed process model was successful in predicting performance in sustained attention tests. In particular, perceptual and mental operation speed consistently showed to be strong predictors. This is not surprising, as the simultaneous presentation of many stimuli is a typical feature of these tests [16], which makes an efficient perception and processing especially beneficial. Moreover, a fast perception and processing allows a quick shift to the next item or even a preprocessing of the upcoming items [62]. Furthermore, there was a trend towards a small influence of motor speed on performance in both studies (Study 1: *p* = .13, Study 2: *p* = .08). Yet, the present sample was young and highly skilled in using computers, which could account for the small variance and low reliability of the measure in both studies [63]. Thus, the role of motor speed could possibly be larger for older participants or for participants who are less familiar with a computer keyboard [64]. Moreover, motor speed was assessed using a computerized task, but only one of the applied sustained attention tests was computerized—the electronic version of the d2-R. Hence, it is conceivable that motor speed as assessed in the current studies could not fully capture the motor processes required in paper–pencil tests. In line with this assumption, descriptively, the influence of motor speed on sustained attention test performance slightly dropped after the electronic d2-R was excluded from the structural equation models. 

Unexpectedly, item shifting, assessed as item shifting speed in Study 1 and as item shifting costs in Study 2, did not predict performance in sustained attention tests. Theoretical considerations and earlier research [16,17] pointed towards a special role of the self-paced presentation mode, indicating that the deliberate shifting from one item to another represents a characteristic demand of sustained attention tests. Indeed, in our two studies, whenever the test-takers were required to immediately shift from one item to another, performance was considerably impeded compared to conditions with longer intervals between successive items. However, the retest reliability of the applied item shifting measures was below .50 in both studies. This insufficiently low reliability could either be due to an inappropriate measurement or because item shifting represents a flexible and dynamic process, and thus, a state rather than a trait.

Finally, it is conceivable that the self-paced presentation mode affects several, possibly every sub-component involved when participants work through a test: Indeed, the self-paced mode demands a permanent deployment of mental effort and the enduring organization of the sub-components of sustained attention tests. This organization of sub-components, namely coordination, has repeatedly been suggested as a critical mechanism of sustained attention tests [15,65,66]. Moreover, it has successfully been extracted and its role for test performance has been demonstrated [66]. Therefore, the permanent deployment of mental effort in self-paced sustained attention tests could go along with an enhanced coordination demand which could likely have an impact on several sub-components beyond only the shifting from one item to another. 

### 4.2. Predicting Higher Cognitive Abilities

It was expected that the proposed process model would be insufficient to explain a large amount of variance in more complex cognitive abilities like reasoning or working memory span, because they involve several additional processes [30,33]. In Study 1, it was demonstrated that the process model performed poorly in predicting test scores in working memory span tasks, confirming discriminant validity. This was in line with our expectations, as tests of working memory span measure the ability to store, maintain and retrieve information from working memory [31,32,33]. Thus, while they also involve perceptual and motor demands, speed in these processes should only be indirectly related to test performance.

Unexpectedly, in Study 1, the sub-components explained a substantial amount of variance in reasoning tests. While it was anticipated that perceptual processes play a role in these tests (see also References [36,54]) we expected the proposed process model to be insufficient to account for the complex mental operations required in reasoning tasks and hence, insufficient to explain a majority of variance in reasoning performance. We believe that this result is partly due to the comparatively complex task in the modified d2 and due to an unusually high correlation between sustained attention and reasoning tests in Study 1. This high correlation could have been caused by a late presentation of the reasoning tests in a four-hour long test session. Under such circumstances, the validity of the reasoning tests might have shifted from measures of reasoning to good indicators of sustained attention. In Study 2, in accordance with our expectations, the process model explained a comparatively small, but still robust amount of variance in reasoning performance. Again, perceptual speed showed to be a strong predictor of reasoning performance, while the influence of mental operation speed was much smaller. Furthermore, note that the factor loadings of the reasoning tasks on the reasoning factor were relatively low compared to the factor loadings of the sustained attention tests on the sustained attention factor, which led to an increased amount of explained variance for reasoning relative to sustained attention. Altogether, discriminant validity of the process model with regard to higher cognitive abilities could, by and large, be confirmed.

Beyond discriminant validity, the investigation of the relationship between the postulated sub-components and reasoning may also have implications for the study of the processes involved in reasoning performance, i.e., the tasks which are considered the best indicator of g. In line with earlier influential research that related individual differences in the speed and efficiency of information processing to g [27,36,67,68,69,70], we found consistent and substantial associations between perceptual speed and performance in reasoning tasks in both studies. Moreover, in Study 1, using a relatively complex modified d2 (including three simultaneously relevant stimuli), mental operation speed was a good predictor of reasoning performance, while the association between mental operation speed and reasoning was much smaller as a simpler modified d2 (presenting only one stimulus at a time) was applied in Study 2. A possible explanation could be that the more complex mental operations required in the first version of the modified d2 might have more effectively captured the (still much more complex) mental operations that are necessary to solve reasoning tasks (see also References [57,58,59]). However, note that, as discussed above, measures of reasoning and sustained attention were also unexpectedly highly correlated in that study.

### 4.3. Limitations

As mentioned earlier, the current samples were not representative with regard to education, age, and sex, as the samples consisted of highly educated, mostly female and young students. Thus, the results cannot be generalized to other samples; the same applies to other tests and tasks. With regard to sample size, a statistical power analysis with simulated data was conducted in Mplus [71]. For both studies, an 80% power to detect significant effects was given for relatively large effects (above .40). Therefore, we should not draw too strong a conclusion about the statistical significance of smaller or moderate effects. However, note that, beyond the significance level, the size of the effect as well as the amount of variance explained in the respective cognitive ability was of special interest in the current study. 

Moreover, the correlation between sustained attention and reasoning tests was higher in the present samples than in earlier studies, which report moderate correlations (for a meta-analysis, see Reference [56] which reports a correlation of *r* = .29). Sustained attention tests have been shown to correlate higher with reasoning than other attention tests, which is interpreted in terms of higher cognitive demands due to a permanent mental effort [11,17]. Nevertheless, even with regard to other studies investigating the relationship between sustained attention and reasoning tests, the observed correlation was above comparative studies and samples. This could be attributed to the length of the test sessions, especially regarding the four-hour test session in Study 1. Anyhow, test sessions in the reference studies were shorter but still several hours long, and thus, the observed result can only partly be attributed to the high endurance demand imposed by the long test session.

Finally, our approach of investigating individual differences in the postulated sub-components of sustained attention tests involved measures of perceptual, mental operation, motor and item shifting speed. However, these sub-components are not necessarily independent or strictly serial [72,73,74] and even relatively simple cognitive tasks include several of them [75]. Therefore, we specified regression models in order to account for shared variance between tasks with overlapping processes. Nevertheless, even using this approach, we might have not fully disentangled the shared processes between the indicators of the postulated sub-components.

## 5. Conclusions

The present study is, to the best of our knowledge, the first to propose and test a process model of sustained attention tests. In two studies, the convergent validity of the process model was demonstrated, while its discriminant validity with regard to higher cognitive abilities was by and large confirmed. It was demonstrated that two main sub-components, namely perceptual and mental operation speed explained a large amount of variance in sustained attention tests. Moreover, in both studies, there was a trend towards a small influence of motor speed on performance. However, a proposed sub-component of item shifting, which was introduced into the model in order to take the self-paced mode of these tests into account, could not be shown. Thus, for now, the impact of the self-paced mode on information processing in cognitive ability tasks remains to be elucidated. Altogether, the present paper may have taken an important first step towards a deeper understanding of the sub-components that drive sustained attention performance.

## Figures and Tables

**Table 1:** Means, standard deviations, and reliability estimates of the measures in Study 1.

Tests/Scores		*M*	*SD*	*r_tt_*
Speed in the Sub-Components				
	Inspection Time (perception)	63.1 ^a^	25.4	-
	Reaction Time *_modified d2_* (simple mental operation)	1321.5 ^a^	349.3	.89 ^c^/.99 ^d^
	Simple Reaction Time (motor reaction)	242.8 ^a^	30.7	.55 ^c^/.60 ^d^
	Response-Stimulus Interval *_modified d2_* (item shifting)	614.5 ^a^	50.9	.41 ^c^/.51 ^d^
Sustained Attention Tests				
	Figural (d2-R electronic version)	222.4 ^b^	40.2	.98 ^d^
	Numerical (Revision Test)	388.1 ^b^	66.7	.96 ^d^
	Verbal (BIS UW/CW/PW)	31.0/22.7/12.6 ^b^	7.1/6.3/2.7	.96/.93/.80 ^d^
Reasoning Tests				
	Figural (matrices, I-S-T 2000 R)	10.5 ^b^	3.0	.61 ^d^
	Numerical (number series, I-S-T 2000 R)	13.2 ^b^	5.1	.90 ^d^
	Verbal (verbal analogies, I-S-T 2000 R)	11.5 ^b^	2.9	.61 ^d^
Working Memory Span Tasks				
	Figural (symmetry span)	15.3 ^b^	4.2	.56 ^d^
	Numerical (operation span)	24.0 ^b^	5.9	.74 ^d^
	Verbal (reading span)	21.1 ^b^	5.8	.67 ^d^

Notes: ^a^ Average speed in ms, ^b^ Number of correct items (minus confusion errors for sustained attention tests), ^c^ Retest reliability, ^d^ Cronbach’s α.

**Table 2:** Means, standard deviations, and reliability estimates of the measures in Study 2.

Tests/Scores		*M*	*SD*	*r_tt_*
Modified d2				
	Reaction Time *_force-paced, single stimuli_*	634.0 ^a^	95.5	.68 ^c^ –.94 ^d^
	Reaction Time *_self-paced, single stimuli_*	702.2 ^a^	96.3	.74 ^c^ –.92 ^d^
	Reaction Time *_force-paced, three stimuli_*	622.6 ^a^	85.0	.89 ^c^ –.95 ^d^
	Reaction Time *_self-paced, three stimuli_*	705.3 ^a^	94.0	.85 ^c^ –.97 ^d^
Sub-Components				
	Inspection Time (perceptual speed)	53.7 ^a^	27.8	.58 ^c^
	Reaction Time *_mean single stimuli_* (simple mental operation)	668.1 ^a^	91.2	.74^c^ –.96 ^d^
	Simple Reaction Time (motor speed)	239.6 ^a^	26.3	.57 ^c^ –.75 ^d^
	Item shifting *_reaction time difference (pace)_*	75.5	49.6	.46 ^c^ –.80 ^d^
Sustained Attention Tests				
	Figural (d2-R electronic version)	220.1 ^b^	33.9	.98 ^d^
	Numerical (Revision-Test)	390.0 ^b^	74.0	.96 ^d^
	Verbal (BIS UW/CW/PW)	34.8/25.2/14.9 ^b^	8.7/5.7/2.5	.95/.95/.83 ^d^
Reasoning Tests				
	Figural (matrices, I-S-T 2000R)	11.4 ^b^	2.9	.60 ^d^
	Numerical (number series, I-S-T 2000R)	13.2 ^b^	4.2	.83 ^d^
	Verbal (verbal analogies, I-S-T 2000R)	11.9 ^b^	3.1	.67 ^d^

Notes: ^a^ Average speed in ms, ^b^ Average number of correct items (minus confusion errors for sustained attention tests), ^c^ Retest reliability, ^d^ Cronbach’s α.

## Footnotes

1 Trials after an RSI of 4000 ms were excluded, because the prolonged RSI was introduced to represent an RSI which is so long that it does not further improve performance [49,50].

2 Mauchly’s sphericity test indicated a violation of the sphericity assumption for the factor RSI (*χ*2(65) = 210.992, *p* < .001). Therefore, the Greenhouse–Geisser correction was used to adjust degrees of freedom for the main effect of RSI (ε = 0.722).

3 The assumption of multivariate normality could not be confirmed (multivariate kurtosis = 37.441, c.r. = 6.726, *p* < .001). A Bollen–Stine bootstrap procedure (1000 samples) was conducted to obtain a corrected *p*-value for the χ2-test.

4 The assumption of multivariate normality could not be confirmed (multivariate kurtosis = 37.074, c.r. = 6.444, *p* < .001). A Bollen–Stine bootstrap procedure (1000 samples) was conducted to obtain a corrected *p*-value for the χ2-test.

5 The assumption of multivariate normality could not be confirmed (multivariate kurtosis = 34.188, c.r. = 8.656, *p* < .001). A Bollen–Stine bootstrap procedure (1000 samples) was conducted to obtain a corrected *p*-value for the χ2-test.

6 The assumption of multivariate normality could not be confirmed (multivariate kurtosis = 25.676, c.r. = 5.349, *p* < .001). A Bollen–Stine bootstrap procedure (1000 samples) was conducted to obtain a corrected *p*-value for the χ2-test.

7 The assumption of multivariate normality could not be confirmed (multivariate kurtosis = 24.943, c.r. = 5.523, *p* < .001). A Bollen–Stine bootstrap procedure (1000 samples) was conducted to obtain a corrected *p*-value for the χ2-test.

## Appendix A

**Table A1:** Correlation matrix of the measures in Study 1.

Tasks		2.	3.	4.	5.	6.	7.	8.	9.	10.	11.	12.	13.	14.	15.
Sub-Components															
	1. Inspection Time Task	.35 **	.19	−.08	.45 **	.35 **	.17	.22 *	.12	.34 **	.35 **	.14	.24 *	.14	.09
	2. Reaction Times *_modified d2_*		.42 *	−.24 **	.65 **	.64 **	.27 **	.25 *	.27 **	.35 **	.47 **	.37 **	.20	.04	−.14
	3. Simple Reaction Time			−.05	.26 *	.18	.12	.20 *	.12	.26 *	.17	.19	.17	.04	.05
	4. Item Shifting Speed				−.30 **	−.14 **	−.03	−.07	−.18 *	−.02	−.11	−.05	−.01	−.00	.08
Sustained Attention															
	5. d2-R (electronic version)					.62 **	.21 *	.32 **	.33 **	.37 **	.43 **	.27 **	.29 **	.17	.18
	6. Revision Test						.24 *	.35 **	.42 **	.27 **	.50 **	.25 *	.17	.17	.08
	7. BIS CW							.57 **	.42 **	.28 **	.11	.39 **	.06	.16	.27 **
	8. BIS PW								.47 **	.40 **	.41 **	.36 **	.23 *	.22 *	.32 **
	9. BIS UW									.22 *	.20 *	.26 **	.11	.06	.20 *
Reasoning															
	10. I-S-T 2000 R Matrices										.37 **	.31 **	.08	.17	.13
	11. I-S-T 2000 R Number Series											.32 **	.23 *	.14	.07
	12. I-S-T 2000 R Analogies												.25 *	.18	.19
Working Memory Span															
	13. Figural Span													.24 *	.22 *
	14. Numerical Span														.48 **
	15. Verbal Span														

Notes: *N* = 100–103, due to excluded data in some tasks (pairwise deletion). * *p* < .05, ** *p* < .01.

## Appendix B

**Table A2:** Correlation matrix of the measures in Study 2.

Tasks		2.	3.	4.	5.	6.	7.	8.	9.	10.	11.	12.
Sub-Components												
	1. Inspection Time Task	.26 *	.11	.01	.26 *	.34 **	.20	.24 *	.17	.17	.28 **	.16
	2. Reaction Time *_modified d2_*		.44 **	−.12	.68 **	.56 **	.43 **	.42 **	.39 **	.04	.43 **	.08
	3. Simple Reaction Time			−.12	.28 **	.25 *	.10	.04	.17	.01	.13	.20 *
	4. Item Shifting Costs				.00	−.02	−.10	.07	.04	−.08	−.14	−.02
Sustained Attention												
	5. d2-R (electronic version)					.57 **	.41 **	.26 **	.41 **	.23 *	.39 **	.21 *
	6. Revision Test						.56 **	.47 **	.57 **	.25 *	.45 **	.28 **
	7. BIS CW							.51 **	.62 **	.23 *	.36 **	.30 **
	8. BIS PW								.38 **	.10	.24 *	.10
	9. BIS UW									.18	.35 **	.32 **
Reasoning												
	10. I-S-T 2000 R Matrices										.35 **	.44 **
	11. I-S-T 2000 R Number Series											.33 **
	12. I-S-T 2000 R Analogies											

Notes: *N* = 94–100, due to excluded data in some tasks (pairwise deletion). * *p* < .05, ** *p* < .01.
